# Extraction of pumpkin peel extract using supercritical CO_2_ and subcritical water technology: Enhancing oxidative stability of canola oil

**DOI:** 10.1007/s13197-020-04624-x

**Published:** 2020-07-09

**Authors:** Azadeh Salami, Narmela Asefi, Reza Esmaeilzadeh Kenari, Mehdi Gharekhani

**Affiliations:** 1 Department of Food Science and Technology, Tabriz Branch, Islamic Azad University, Tabriz, Iran; 2 Department of Food Science and Technology, Sari Agricultural Sciences and Natural Resources University, Mazandaran, Iran

**Keywords:** Pumpkin peel extract, Antioxidant activity, Phenol, Carotenoid, Canola oil

## Abstract

In this study, subcritical water extraction (SWE) and the supercritical fluid extraction (SFE) methods were used for the extraction of pumpkin peel extract. Total phenolic content and carotenoid compounds of extracts were measured. The extracts were added to canola oil at a concentration of 400 ppm and were stored at 30 °C for 60 days. The peroxide, carbonyl and acid values of the oil samples were measured, then compared with 100 ppm of tert-butylhydroquinone (TBHQ) synthetic antioxidants. The results showed that the total phenol content of obtained extract by SFE (353.5 mg GA/100 g extract) was higher than by SWE (213.6 mg GA/100 g extract), while the carotenoid content was higher for obtained extract by SWE (15.22 mg/100 g extract) compared to SFE (11.48 mg/100 g extract). The result of oil oxidation showed that the oxidative stability of the oil containing the mixed extract (SFE–SWE) is higher than the separate extract, consequently showing higher performance in preventing oil oxidation compared to TBHQ.

## Introduction

Herbal extracts are a variety of natural antioxidants, offering a unique range of health benefits. The secondary metabolites of plants, such as phenolic and carotenoid compounds, are valuable sources of antioxidants extracted from different parts of the plant, including leaves, seeds and, peels (Dabbou et al. 2017).

Pumpkin is a cultivar of a squash plant, most commonly *Cucurbita pepo*, cultivated because of the nutritional value of the pulp and its seeds and used in the production of syrups, jellies, jam and purees (Provesi et al. 2011). Pepo peel is usually removed from the fruit before use, and previous studies showed that *C. pepo* peel is a rich source of antioxidant compounds (Tavakoli et al. 2017). Also, pumpkin peel is a source of pectin, minerals, vitamins and other compounds beneficial to the human health (de Carvalho et al. 2012). Pulp and peel of pumpkin contain high levels of carotenoids, natural molecules containing terpenes with 40 carbon atoms creating yellow–red colors in flowers, leaves and fruits. They are categorized in carotenes and xanthophylls (Oliver and Palou 2000; Kehili et al. 2017).The application of conventional solvent extraction methods is not recommended due to the dangers of organic solvent residues in the extract, time-consuming extraction, damage to the environment, and the active compounds of the plant. Today, novel extraction techniques, such as, microwave, ultrasound, SWE and SFE, as green technologies, have replaced older methods (de Andrade Lima et al. 2018).

SFE is a method that basically uses carbon dioxide, as a solvent, for the extraction and is used as a clean method for extracting bioactive compounds from plant lesions, such as fruits and vegetables. In this method, the extraction of compounds is similar to the conventional extraction methods; however, the fluids are in the supercritical state of viscosity and surface tension, like gases, and density, while they have dissolving power like fluids (Kehili et al. 2017; de Andrade Lima et al. 2018). Similar properties make carbon dioxide an ideal fluid for extracting compounds in shorter time periods with greater efficiency than liquid solvents (de Andrade Lima et al. 2018).

The oxidation of fats, as a result of the reaction between oxygen and unsaturated fatty acids, causes a lot of problems for the oil factories. Not only does oxidation result in the lower quality of oils and fats due to chemical-corrosive reactions, but it also leads to the production of peroxyl and hydroxyl free radicals and reactive oxygen species, resulting in heart disease, aging and mutagenesis (Islam et al. 2018). Canola oil is preferable to other vegetable oils considering its high amounts of unsaturated fatty acids. However, due to its low thermal stability at high temperatures, it is necessary to increase its oxidative stability by adding antioxidants (Farahmandfar et al. 2015).

Antioxidants are substances used to prevent oxidation in human bodies and food products. Synthetic antioxidants are added as additives to foods to prevent spiky reactions, revealed to their high efficiency, low prices and abundance. The most important synthetic antioxidants used as preservatives for increasing the stability of vegetable oils in the food industry include butylated hydroxyl toluene (BHT), tert-Butyl hydroquinone (TBHQ) and butylated hydroxyl anisole (BHA). With regard to the identification of the effects of synthetic antioxidants on the liver and the development of cancer, consumers' desire to use natural antioxidants has increased noticeably (Agregán et al. 2017; Kehili et al. 2017).

The objective of the present work is to propose a cheap and environmentally friendly method for extraction of pumpkin peel extract containing the highest value of carotenoid and phenol using subcritical water and supercritical CO_2_ extraction methods and their effect on the stability of canola oil.

## Materials and methods

### Materials and chemical reagents

Bleached and odor-neutralized canola oil, without antioxidants, was obtained from Beheshahr Agricultural Industrial Complex (Behshahr, Iran). All materials used in the research were of analytical grade from Sigma- Aldrich Company (St. Louis, the USA). Pumpkin of *Cucurbita pepo* Styarica variety was purchased from a local market in Sari (Mazandaran Province, Iran) in autumn 2018. All of the chemicals and solvents used were analytical grade and provided from Sigma-Aldrich (India).

### Pumpkin peel extract preparation

Pumpkins were washed with cold water after entering the laboratory and their peels were removed manually. The thickness of the peel was 1.0 ± 0.2 cm. The pumpkin peel was dried in an oven at 40 °C and powdered with a particle size of 2 mm (Cuco et al. 2019).

### Subcritical water extraction(SWE)

First, 12 g pumpkin peel was put inside the extractor with glass beads. Then, the extractor was installed on the heater. Water as a solvent was pumped at a flow rate of 1 ml/min using a HPLC (High Performance Liquid Chromatography) pump to achieve the desired pressure. The pressure was adjusted by a heat regulator. Then, the water was heated to a working temperature using a pre-heating device. Extractor temperature was measured to ensure that the desired temperature was reached. The temperature, time and pressure used were 120 °C, 3 h and 5 MPa, respectively. The extraction was carried out under the optimal condition of the recovery of carotenoid and total phenolic compounds. The extracted solution was collected in vial and stored in a refrigerator (Setyorini et al. 2018).

### Supercritical fluid (CO2) extraction (SFE)

First, 12 g pumpkin peel was put inside the extractor with glass beads. Then, the extractor was installed on the heater. The carbon dioxide fluid was pumped as a solvent at a flow rate of 15 ml/min using a HPLC pump to achieve the desired pressure. Then, ethanol: water (80:20) was pumped using a HPLC pump at a rate of 0.25 ml/min. A mixture of carbon dioxide and ethanol: water (80:20) was entered the extractor. The pressure was adjusted by a heat regulator. The water was heated to a working temperature using a pre-heating device. Extractor temperature was measured to ensure that the desired temperature was reached. The temperature, time and pressure used were 60 °C, 3 h and 25 MPa, respectively. The extraction was carried out under the optimal condition of the recovery of carotenoid and total phenolic compounds. The extracted solution was collected in vial and stored in a refrigerator (Setyorini et al. 2018).

### Determination of total phenolic and carotenoid content

The total phenolic content (TPC) of extract was determined according to the Folin–Ciocalteu method, as described by Setyorini et al. (2018). Gallic acid was used as the standard and results were calculated on the basis of calibration curve of gallic acid and expressed as gallic acid equivalents (mg GAE/100 g). β-Carotene content in the extract was measured using spectrophotometer at a wavelength of 450 nm, as described by Setyorini et al. (2018).

### HPLC-analysis of carotenoid

Carotenoids were analyzed according to the method (Machmudah et al. 2012) and (Shi et al. 2010) with slight modifications. Separation was carried out using HPLC (Shimadzu HPLC-10 AT) with XDB-C18 column (5 μm, 1.4 × 150 mm) and UV–vis detector (monitored at 470 nm. The sample was injected in 20 µunits. A mixture of methanol/ methyl tert-butyl ether/water with different ration of 81:15:4, v/v/v (A) and 4:92:4, v/v/v (B) was used as a mobile phase at flow rate of 1.5 ml/min. Gradient elution program was as follows: 0–60.0 min, solvent B increasing from 0 to 80%; 60.0–65.0 min, solvent B increase to 100%; 65.0–70.0 min, solvent B decrease to 0%; 70.0–80.0 min, isocratic with 0% B. The amount of carotene in the extract was compared based on the retention time and peak area of the standard sample. In other words, peaks were determined based on the retention time and UV absorption patterns of the standards. Because *cis*-isomer standards were not available, the quantification of b-carotene isomers was carried out by applying the same response factor as all-trans-b-carotene.

### HPLC-analysis of total phenolic compounds

The phenolic compounds of the pumpkin peel extract were analyzed according to the method of (Uddin et al. 2014) with slight modifications. Separation was carried out using HPLC (Shimadzu HPLC-10 AT) with XDB-C18 column (5 μm, 1.4 × 150 mm) and UV–vis detector. The mobile phase included of acetonitrile (A), acetic acid solution at pH 3.0 (B), and methanol (C). Gradient chromatography was run as follows: 0 min, 5%A:95%B; 10 min, 10%A:80%B:10%C; 20 min, 20%A:60%B:20%C and 30 min, 100%A. The flow rate was at 1 ml/min and the injection volume was 20 μl. For UV detection, the wavelength was optimized to phenolic compounds at their maximum absorbance wavelengths (280 nm). The phenol content of the extract was compared based on the retention time and peak area of the standard sample. In other words, peaks were determined based on the retention time and UV absorption patterns of the standards.

### Antioxidant activities of pumpkin peel extract

#### Free radical scavenging DPPH

First, 2.7 ml of the freshly prepared DPPH solution (6 × 10^–5^ mol/l) was mixed with 0.3 ml of 4 concentrations of 100, 200, 300 and 400 ppm of extract and 100 ppm of synthetic anti-oxidant TBHQ, as the positive control. Then, the resultant mixture was stirred vigorously and kept in dark for 1 h. Finally, the absorbance was read at 517 nm and calculated according to the following equation:

$$ {\text{DPPH inhibition }}\left( \% \right) \, = \left( {A_{blank} - A_{sample} } \right)/A_{blank) \times 100} $$

where, A_sample_ andA_blank_ are the absorption of extract and control without extract, respectively (Esmaeilzadeh Kenari et al. 2014).

#### Ferric reducing ability power (FRAP)

In brief, 2.5 ml of the extract solution was combined with 2.5 ml of sodium phosphate buffer (200 mmol/l) and 2.5 ml of 1% ferricyanide and the mixture was incubated for 20 min at 50 °C. Then, 2.5 ml of 10% v/v trichloroacetic acid was added to the mixture, after which the resultant mixture was centrifuged at 116 g for 8 min (HERMEL Z 9 200A). 5 ml of the top solution was combined with 5 ml of the deionized water and 1 ml of iron chloride (0.1%). Finally, the absorbance of the solution was read at 700 nm. Synthetic antioxidant TBHQ was used as the positive control (100 ppm) (Esmaeilzadeh Kenari et al. 2014).

#### Preparation of oil

To examine the antioxidant activity of the obtained extracts, different extracts were added to canola oil at a concentration of 400 ppm and be compared with 100 ppm of the synthetic antioxidant TBHQ. The oil samples were stored at thermal conditions of 30 °C for 60 days. The oil analysis was performed at different days of 0, 15, 30, 45, and 60 (Sayyad and Farahmandfar 2017). An antioxidant-free oil sample was also considered as the control.

### Chemical properties of canola oil

#### Peroxide value

The peroxide value was evaluated based on the AOAC method, No. 33/965(Chemists 1990). First, 5 g of canola oil was dissolved in 10 ml of trichloromethane. Then, 15 ml of acetic acid and 1 ml of potassium iodide saturated solution were added and gently stirred and stored for 5 min in the dark. After the incubation time was completed, 75 ml of the distilled water was added to it and severely mixed up. Finally, it was normalized with sodium thiosulfate (0.01 N). Finally, the peroxide value was calculated based on the following equation in terms of mEq of oxygen/kg oil:

$$ {\text{PV}} = \frac{{\left( {{\text{V}}2 - {\text{V}}1} \right) \times {\text{N}} \times 1000}}{{\text{m}}} $$

where, V_2_ and V_1_ are the sample and control titration numbers, respectively, N is the sodium thiosulfate normality and m is the sample weight in gram.

#### Carbonyl value

First, 1 kg of 2 propanol and 0.5 g sodium borohydride were refluxed for 1 h, in order to remove additional carbonyls in the solvent. Then, 2 and 4 di-nitrofenylhydrazine (DNPH) of 50 g was dissolved in 100 ml solvent, containing 3.5 ml of chloride acid 37%. Canola oil reached a volume of 10 ml at the rate of 0.04–1 g through the addition of a solvent including trinylpyrrole (0.4 mg/ml). After that, 50 μm solution 2 and 4 decadienal were prepared in 2-propanol. 1 ml of the oil sample was combined with 1 ml of DNPH and heated to 40 °C for 20 min, and then, after adding 8 ml of potassium hydroxide (2%), it was cooled in bath water. Finally, the sample absorption was read after 5 min centrifugation at 2000 g at 420 nm (Endo et al. 2001).

#### Acid value

Firstly, 10 g of the oil samples were weighed in the Erlenmeyer and dissolved in 50 ml of chloroform: ethanol solvent (50:50). Then, a few drops of phenolphthalein were added as reagent to it and titrated with normal potassium hydroxide 0.1. Finally, the acid value was obtained according to the following equation (Firestone 1973).

$$ {\text{Acid value}} = \frac{{{\text{V}} \times {\text{C}} \times 56.11}}{{\text{m}}} $$

where, m is the weight of the oil in grams, V is the amount of potassium hydroxide consumed in milliliters, and C is the concentration of potassium hydroxide in moles per liter.

#### Statistical analysis

The statistical analysis of the data, obtained from the extraction section, was performed using t-test. For chemical properties of canola oil, a completely randomized design with one-way ANOVA was used. A significant statistical difference was found between the means at the 95% probability level using Duncan's multiple range tests. The software used was SPSS version 20. In order to reduce the error, all tests were performed in triplicate.

## Results and discussion

### The amount of phenolic and carotenoid compounds of the extracts

The results of measuring the amounts of phenolic and carotenoid compounds of the extracts are shown in Table 1. It was observed that the SFE method used to extract phenolic compounds was more effective than the SWE. The simultaneous application of temperature and pressure in the SFE played an important role in increasing the strength of carbon dioxide solubility, effectively increasing the extraction of phytochemicals and nutrients of pumpkin peel (Prado et al. 2014). High pressure led to the breakdown of the cell walls of the plant and strong chemical interactions between carbohydrate and lipid compounds with the wall, ultimately providing the easy exit of carotenoids from the extraction bed (Khajeh 2011). Carotenoids have high molecular weight and lower polarity (de Andrade Lima et al. 2018). The total carotene extracted in the SWE method was higher than that of the SFE method. Hamdan et al. (2008) showed that the carotenoid and chlorophyll pigments in the SWE method were higher than those in the SFE method, which was consistent with the results of the present study.

**Table 1:** The amount of phenolic and carotenoid compounds of the extracts

Samples	Total phenol (mg GA/100 g E)	Total carotene (mg carotene/100 g E)
SWE	213.6 ± 5.87^b^	15.22 ± 2.35^a^
SFE	353.5 ± 8.84^a^	11.48 ± 0.90^b^

*Note* Pumpkin peel extract obtained bysubcritical water extraction (SWE) and supercritical fluid extraction (SFE)

Values (Mean ± SD, n = 3) in the same column with different letters are significantly different (P < 0.05)

### Effective compounds profiles of the extracts

The profiles of carotene compounds of extracts are shown in Table 2. As observed, many compounds were identified by HPLC, and the value of detected carotene compounds in the extract produced by SWE was more than that produced by SFE. β-Carotene is also an important compound as an anti-oxidant and vitamin A precursor (Hamdan et al. 2008). Lutein is one of the most important carotenoids in the diet. It is inexpensive and has antioxidant activity (Goto and Watanabe 2012).

**Table 2:** The profiles of carotenoid compounds of extracts was obtained by two different methods

Rt (min)	SWE	SFE	Compound (%)
11.5	4.77	0.22	Neoxanthin
12.0	4.67	0.27	Violaxanthin
15.2	8.78	2.49	Lutheoxanthin
15.4	10.79	3.97	Lutein
17.3	2.25	0.09	Unknown
17.8	3.06	0.06	Zeaxanthin
32.3	1.22	0.14	13*-cis*-β-carotene
34.8	0.17	0.05	α-Carotene
35.2	0.14	0.09	Unknown
38.7	23.13	25.15	α-Crypthoxanthin
39.6	1.62	1.36	β-Carotene
40.1	2.41	0.05	9-*cis*-β-carotene
44.2	12.76	24.01	β-Crypthoxanthin
46.0	1.51	0.05	Lycopene
–	77.28	57.99	Total carotenoid

*Note* Pumpkin peel extract obtained by supercritical fluid extraction (SFE) and subcritical water extraction (SWE)

Quantitative and qualitative results regarding the number of phenolic compounds of pumpkin peel extracts are shown in Table 3. As can be seen, the number of phenolic compounds extracted by the SFE was higher. Vanillic acid, *p*-coumaric acid, and sinapic acid were the most important phenolic compounds in the pumpkin peel (Dragovic-Uzelac et al. 2005) hydroxycinnamic acids (such as caffeic, *p*-coumaric, ferulic, and sinapic acids) are compounds in the cell walls and they are responsible for preventing pathogens from entering the plant (Peričin et al. 2009).

**Table 3:** The profiles of phenolic compounds of extracts was obtained by two different methods

Rt (min)	SFE	SWE	Compound (%)
6.5	0.02	6.7	Protocatechuic
9.2	11.9	20.8	*p*-Hydroxybenzoic
12.3	11.5	1.27	*p*-Hydroxybenzaldehyde
16.2	4.13	5.61	Vanillic
16.9	21.6	2.32	Caffeic
17.11	0.03	0.06	Syringic
17.3	8.02	1.7	Trans-*p*-Coumaric
19.2	7.82	3.27	Ferulic
20.4	0.04	3.85	Trans-Sinapic
–	65.06	45.58	Total phenolic

*Note* Pumpkin peel extract obtained by supercritical fluid extraction (SFE) and subcritical water extraction (SWE)

### Antioxidant activities of pumpkin peel extract

**Fig.1:**
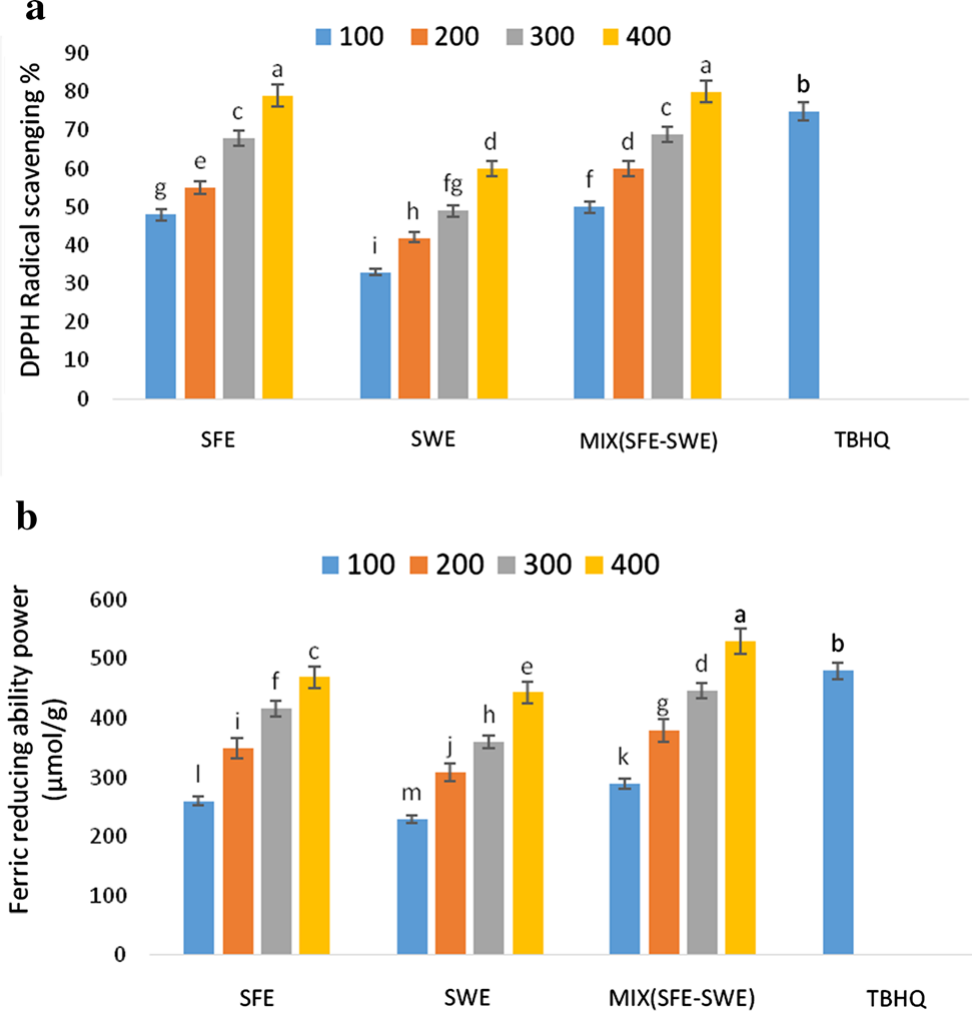
DPPH radical scavenging (**a**) and iron reducing (**b**) of different concentrations of extracts (100–400 ppm) and TBHQ (100 ppm) *Note* Pumpkin peel extract obtained by method of supercritical fluid extraction (SFE) and subcritical water extraction (SWE). MIX(SFE–SWE) is a mixture of SFE and SWE extracts

DPPH free radical scavenging is one of the fastest methods for determining the capacity of hydrogen donation of chemicals, and so for evaluating their antioxidant activity. When the DPPH molecule encountered a radical proton, its purple color disappeared quickly (Agregán et al. 2017). In the method of reducing iron, free radicals are neutralized either by electron transport or the disposal of hydrogen atoms. These methods are easy and inexpensive, therefore, frequently used in factories (Hiranvarachat and Devahastin 2014). The results of measuring the antioxidant activity of the extracts using DPPH radical scavenging and iron-reducing methods are shown in Fig. 1a, b, respectively. As it can be seen, with increased concentrations of extracts, the amount of radical scavenging and iron-reducing increased and a statistically significant difference was seen. Hamdan et al. (2008) showed that antioxidant activity in the SFE-derived extract was more than that in SWE extract, in a good agreement with the results of the present study. The type of extracted compounds affected the antioxidant activity of the extracts. Shi et al. (2013) reported that the antioxidant activity of carotene extracts was related to their fractional components. The type of extracted compounds affected the antioxidant activity of the extracts. According to Shi et al. (2013), the antioxidant activity of carotene extracts was associated with their fractional components. Cis isomers in the extract such as 9-*cis*-*β*-carotene and 13-*cis*-*β*-carotene had higher antioxidant activity than trans-types (Shi et al. 2013). The combined extracts exhibited higher antioxidant activity, at the same concentration.

Agregán et al. (2017) reported that the antioxidant activity of herbal extracts in the iron reduction test was related to the concentration of extracts, reporting an increasing trend in scavenging free radicals by increasing the concentration of the extract. The type of compounds in the extract affected their antioxidant activity (Agregán et al. 2017). As observed, the quality of phenolic compounds in the extracts produced from various methods was different. The non-phenolic compounds present in the extract such as molecules of low molecular protein and carbohydrate weights also affected the amount of free radical scavenging (Gallardo et al. 2013).The antioxidant activity of the extracts was related to the value and position of the hydroxyl groups of phenolic compounds. For example, caffeic acid with two hydroxyl groups had higher antioxidant activity than that of *p*-coumaric acid with one hydroxyl group (Masek et al. 2016). The amount of both phenols mentioned in the extract produced with SFE was higher than those in SWE.

The concentration of 400 ppm of the extract was showed the highest antioxidant activity with measuring total phenolic content and antioxidant activity. Then, the pumpkin peel extract with a concentration of 400 ppm was chosen to injecting to the canola oil separately and in a combination form, and compared with 100 ppm of synthetic TBHQ antioxidant.

### Chemical properties of canola oil

#### Peroxide value

**Fig. 2:**
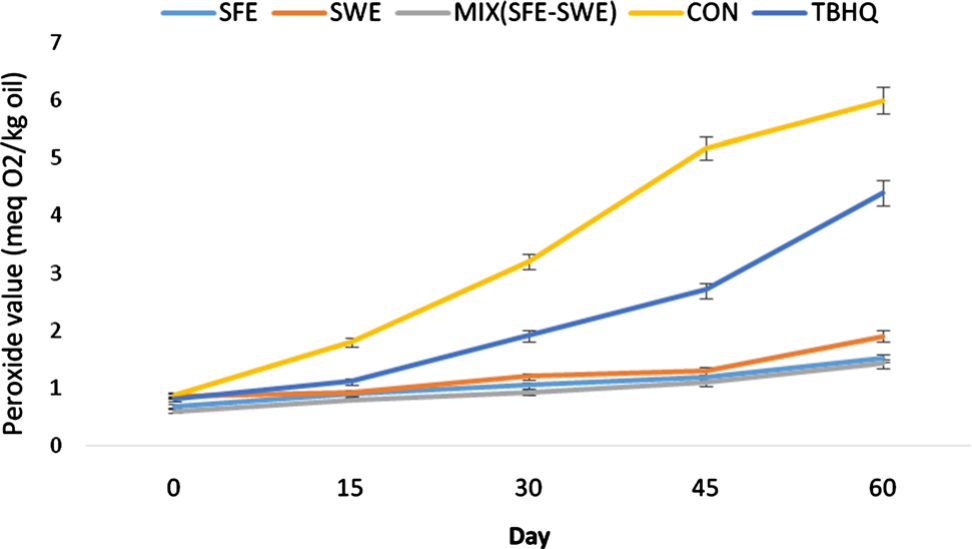
The changes of peroxide value of different oil samples during storage. *Note* Pumpkin peel extract obtained by method of Supercritical fluid extraction (SFE) and Subcritical water extraction (SWE). MIX(SFE–SWE) is a mixture of SFE and SWE extracts

Peroxide value is a very good indicator for evaluating peroxide production at the beginning of the oxidation process. It is equal to the amount of peroxide and hydroperoxide formed during the oxidation process (Zhang et al. 2010; Agregán et al. 2017; Islam et al. 2018). As shown in Fig. 2, over time, the peroxide value increased in all treatments, indicating the formation of hydroxides during the maintenance period (Islam et al. 2018). In the control sample, in 0 to 45th day of the storage period, the amount of peroxide increased from 0.88 to 5.17 meq O_2_/kg oil. In the oil sample containing TBHQ at the end of the maintenance period, the peroxide value was 4.14 meq O_2_/kg oil, higher than that of the extract-containing oil samples. Adding phenolic and carotene extracts of pumpkin peel to canola oil resulted in the increased oxidative stability of the oil. Islam et al. (2018) showed that the use of pomegranate and orange peel extracts in soybean oil and sunflower oil led to the increased oxidative stability of the oil. Herbal extracts caused increased human health because they prevent the oxidation of fats and scavenge-free radicals. Agregán et al. (2017) compared the antioxidant activity of marine algae extract in canola oil with synthetic antioxidant BHT. Over time, peroxide value increased and the control sample had the highest peroxide value.

Peroxide value ranged from 0.68 to 1.52 for the extract produced by the supercritical fluid, 0.85 to 1.90 for the extract produced by the following sub-critical water method and 0.6 to 1.42 for MIX(SFE–SWE). These results indicated that MIX(SFE–SWE) extract showed a higher antioxidant effect than separate extract. The higher oxidation stability of the oil sample containing the combined extract could be attributed to the types of its constituents. Both the SFE and SWE methods were useful in extracting plant's various main components, and each of which showed different functions in preventing the oil oxidation process. Therefore, the combined SFE and SWE extract had been more functional. As shown in Fig. 2, the plant extracts had higher antioxidant activity than synthetic antioxidants, which was consistent with the results of Islam et al. (2018). In addition, the results confirmed that the extracts had the ability to prevent the oxidation of oil in the early stages of oxidation by donating electrons and inhibiting free radicals (Kindleysides et al. 2012; Farvin and Jacobsen 2013; Agregán et al. 2017).

#### Carbonyl value

The results obtained for the value changes of carbonyl of different oil samples are shown in Table 5. As can be seen, carbonyl content increased in all treatments over time, and a significant statistical difference was seen. The lowest increase in the carbonyl content was observed in the oil samples containing combined extracts; besides, the oil containing TBHQ had a higher carbon value than the extract-based oils. Elbadrawy and Sello (2016) examined the antioxidant properties of tomato peel extract in increasing the stability of flaxseed oil during 28 days of storage. Carbonyl value changes were incremental in all samples and the control sample and BHT-containing oil had the highest carbon number, respectively. The olive oil containing petroleum extract of tomato wort had the lowest carbon number. The extract of tomato peel had high antioxidant properties due to phenolic and lycopene compounds. Other research results showed that the amount of phenolic and flavonoid compounds in the peel was higher than seeds and pulp (Stewart et al. 2000). As shown in Table 4, the carbonyl value of SWE extract was slightly higher than that of SFE extract, although the lowest content of carbonyl value was related to the oil sample containingMIX (SFE–SWE) extract.

**Table 4:** The value changes of carbonyl of different oil samples during storage (μmol/g)

Sample	Day				
	0	15	30	45	60
CON	10.51^ Da^	11.36^ Da^	18.79^Ca^	33.80^Ba^	35.64^Aa^
TBHQ	9.46^Db^	10.22^Db^	16.91^Cb^	30.42^Bb^	33.72^Ab^
SFE	8.62^ Da^	9.07^Db^	12.35^Cc^	17.23^Bc^	21.05^Ac^
SWE	9.57^Db^	9.20^Db^	12.52^Ccd^	17.90^Bc^	22.73^Ac^
MIX(SFE–SWE)	7.18^Dc^	7.25^Dc^	9.88^Ce^	15.34^Be^	18.04^Ad^

*Note* Pumpkin peel extract obtained by supercritical fluid extraction (SFE) and subcritical water extraction (SWE). MIX(SFE–SWE) is a mixture of SFE and SWE extracts, and CON is control oil (Without any antioxidant)

Different little letters within the same column indicate significant differences (P < 0.05)

Different big letters within the same row indicate significant differences (P < 0.05)

#### Acid value

The acid value is an indicator of fatty acid hydrolysis. Free fatty acids, formed as a result of the hydrolysis of triglycerides, are an indicator of the rate of oil shrinkage (Islam et al. 2018). Table 5 shows the results of the acid value of different oil samples during the maintenance period. The control sample had the highest acid value. The acid value in TBHQ-containing oil samples was higher than that of the extract containing oil samples. The acid value in the oil samples containing the MIX (SFE–SWE) extract was lower than that in the oil samples containing extracts individually. The extract produced by the SFE, compared to the extract obtained by SWE, showed higher antioxidant properties in reducing the acid value of oil. These results were consistent with those of El-aal and Halaweish (2010), showing that in soybean oil containing ethanolic extracts of orange peel, hydrolysis of fatty acids occurred less than synthetic antioxidants. Also, Lutfullah et al. (2015) showed that formed free fatty acids in soybean oil containing pomegranate peel extract, were less than those in the oil containing synthetic BHT antioxidants. Arawande and Borokini (2015) examined the antioxidant properties of orange peel extract in peanut oil during 14 months of storage at 27 to 30 °C. According to their results, over time, the acid value increased and the sample showed the highest acid value. Furthermore, the antioxidant activity of the extracts was better than that of synthetic antioxidants in reducing the acid value, consistent with the results of this study (Arawande and Borokini 2015). Purwaningsih et al. (2019) showed that the use of banana peel extract led to a reduction in the acid value of vegetable oil samples, due to the presence of phenolic compounds.

**Table 5:** The acid value of different oil samples during storage (mg KOH/g oil)

Sample	Day				
	0	15	30	45	60
CON	0.24^Ea^	0.36^ Da^	0.53^Ca^	1.08^Ba^	1.63^Aa^
TBHQ	0.20^Db^	0.23^Db^	0.39^Cb^	0.70^Bb^	1.17^Ab^
SFE	0.18^Db^	0.20^Db^	0.25^Cc^	0.34^Bc^	0.432^Ac^
SWE	0.19^Db^	0.20^Db^	0.27^Cd^	0.39^Bd^	0.50^Ad^
MIX(SFE–SWE)	0.17^Db^	0.17^Dc^	0.22^Ce^	0.30^Be^	0.36^Ag^

*Note* Pumpkin peel extract obtained by supercritical fluid extraction (SFE) and subcritical water extraction (SWE). MIX(SFE–SWE) is a mixture of SFE and SWE extracts, and CON is control oil (Without any antioxidant)

Different little letters within the same column indicate significant differences (P < 0.05)

Different big letters within the same row indicate significant differences (P < 0.05)

## Conclusion

According to the results of the present study, the use of pumpkin peel extracts, obtained using SFE, showed a strong protective effect against canola oil oxidation during storage, in contrast to those obtained by SWE. In addition, the mixture extract (SFE–SWE) with higher free-radical scavenging and iron-reducing effect, compared to the extracts obtained by SFE and SWE did separately, prolonged the stability of canola oil during storage. The fact that the SFE + SWE extract was more effective could be attributed to the synergistic effects of the extracts as well as the different types of materials extracted by two methods. The findings of this study indicated that the natural antioxidant extract of pumpkin peel, thanks to phenolic and carotene compounds under these conditions, can be used as an alternative to synthetic antioxidants in edible oil refineries.
